# Lysosomal Proteomics Links Disturbances in Lipid Homeostasis and Sphingolipid Metabolism to CLN5 Disease

**DOI:** 10.3390/cells11111840

**Published:** 2022-06-04

**Authors:** Stefano Doccini, Maria Marchese, Federica Morani, Nicola Gammaldi, Serena Mero, Francesco Pezzini, Rabah Soliymani, Melissa Santi, Giovanni Signore, Asahi Ogi, Silvia Rocchiccioli, Katja M. Kanninen, Alessandro Simonati, Maciej M. Lalowski, Filippo M. Santorelli

**Affiliations:** 1 Molecular Medicine–IRCCS Stella Maris, 56128 Pisa, Italy; maria.marchese@fsm.unipi.it (M.M.); nicola.gammaldi@fsm.unipi.it (N.G.); s.mero27@gmail.com (S.M.); asahi.ogi@fsm.unipi.it (A.O.); 2 Department of Biology, University of Pisa, 56126 Pisa, Italy; federica.morani@biologia.unipi.it; 3 Ph.D. Program in Neuroscience, University of Florence, 50121 Florence, Italy; 4 Department of Surgery, Dentistry, Paediatrics and Gynaecology, University of Verona, 37129 Verona, Italy; francesco.pezzini@univr.it (F.P.); alessandro.simonati@univr.it (A.S.); 5 HiLIFE, Meilahti Clinical Proteomics Core Facility, Faculty of Medicine, University of Helsinki, 00014 Helsinki, Finland; rabah.soliymani@helsinki.fi; 6 NEST, Scuola Normale Superiore and Istituto Nanoscienze-CNR, 56127 Pisa, Italy; melissa.santi@sns.it; 7 Fondazione Pisana per la Scienza, 56017 Pisa, Italy; giovanni.signore@unipi.it; 8 Institute of Clinical Physiology-CNR, 56124 Pisa, Italy; silvia.rocchiccioli@ifc.cnr.it; 9 A. I. Virtanen Institute for Molecular Sciences, University of Eastern Finland, 70210 Kuopio, Finland; katja.kanninen@uef.fi; 10 Institute of Bioorganic Chemistry, PAS, Department of Biomedical Proteomics, 61-704 Poznan, Poland

**Keywords:** NCL, CLN5 disease, lysosomes, lysosomal proteomics, trehalose, miglustat

## Abstract

CLN5 disease (MIM: 256731) represents a rare late-infantile form of neuronal ceroid lipofuscinosis (NCL), caused by mutations in the *CLN5* gene that encodes the CLN5 protein (CLN5p), whose physiological roles stay unanswered. No cure is currently available for CLN5 patients and the opportunities for therapies are lagging. The role of lysosomes in the neuro-pathophysiology of CLN5 disease represents an important topic since lysosomal proteins are directly involved in the primary mechanisms of neuronal injury occurring in various NCL forms. We developed and implemented a lysosome-focused, label-free quantitative proteomics approach, followed by functional validations in both *CLN5*-knockout neuronal-like cell lines and *Cln5*^−/−^ mice, to unravel affected pathways and modifying factors involved in this disease scenario. Our results revealed a key role of CLN5p in lipid homeostasis and sphingolipid metabolism and highlighted mutual NCL biomarkers scored with high lysosomal confidence. A newly generated *cln5* knockdown zebrafish model recapitulated most of the pathological features seen in NCL disease. To translate the findings from in-vitro and preclinical models to patients, we evaluated whether two FDA-approved drugs promoting autophagy via TFEB activation or inhibition of the glucosylceramide synthase could modulate in-vitro ROS and lipid overproduction, as well as alter the locomotor phenotype in zebrafish. In summary, our data advance the general understanding of disease mechanisms and modifying factors in CLN5 disease, which are recurring in other NCL forms, also stimulating new pharmacological treatments.

## 1. Introduction

Neuronal ceroid lipofuscinosis (NCL) is a group of inherited, neurodegenerative, lysosomal storage disorders (LSDs) that affects all ages and ethnicities and collectively represents the most frequent form of childhood dementia [1]. Although many advances have been made in a more rapid genetic diagnosis, no treatment other than palliative care is currently available for most forms of NCL but CLN2, in which an enzymatic replacement therapy has been demonstrated to modify the disease trajectories if started early in the course of a disease [2,3]. The lack of efficient therapeutic opportunities derives also from a limited knowledge on the pathogenic mechanisms.

CLN5 disease (MIM: 256731) represents a rare, non enzymatic, late infantile form of NCL caused by mutations in the *CLN5* gene. The gene product is a soluble, yet uncharacterized, lysosomal glycoprotein, which does not share evident homology with other proteins. It putatively interacts with other NCL proteins, including CLN1, 2, 3, 6, and 8 [4,5,6,7]. The function of CLN5 remains elusive, with published data suggesting roles in: endosomal sorting [8], glycoside hydrolase activity [9], and regulation of autophagic flux [10]. Our proteomic investigations of mitochondria derived from *CLN5* knockout cell models and in the brains of *Cln5*^−/−^ mice highlighted a functional link between loss of CLN5p expression and altered lipid metabolism, bioenergetics, and oxidative stress, in line with the activation of autophagy or mitophagy among others [11]. Our data also proposed CLN5 involvement in the early steps of oxidative stress at the pre-symptomatic stage of the disease. In the current work, proteomic analyses on lysosomes isolated from various CLN5 models, together with the multilayer confidence assessment of lysosomal localization, were used to pinpoint previously unknown pathological mechanisms. Our study also revealed several high-confidence lysosomal proteins shown to be dysregulated in other NCL forms [12,13,14,15], further reinforcing the idea of a common pathogenic theme in various forms of the disease [16,17]. The role of CLN5 in neurodevelopment was functionally investigated in vivo using zebrafish morphants, a knockdown model already utilized in various NCL forms [18]. Utilization of CLN5 cellular and animal models, as well as CLN5 patients’ derived cells, allowed the translation of specific experimental findings into a possible therapeutic approach. The prospect to modulate the susceptibility to oxidative stress and lipid metabolism in vitro, and the locomotor behavior in vivo, was assessed with two FDA-approved drugs, enabling one to rescue the CLN5 phenotype and encouraging the use of such readouts for rapid assessment of potential NCL treatments.

## 2. Materials and Methods

### 2.1. Disease Models and Standard Methodologies

Generation of disease models and standard methods to charcaterize in vivo and in vitro models are summarized in Supplementary Methods.

### 2.2. Subcellular Fractionation of Cells and Tissues for Lysosome Enrichment

Isolation of lysosomal fractions from HEK 293T cell lysates and mice cerebral cortex was performed using the Lysosome Enrichment Kit (Thermo Scientific™, Waltham, MA, USA) according to manufacturer instructions for Cultured Cells (protocol 2 with sonication) and Soft Tissue (protocol 4 with polytron tissue tearer), respectively. About 50 mg of cells obtained from four confluent F75 flasks (harvested without trypsin) or 150–200 mg of fresh cerebral cortex homogenized tissue were processed for lysosome enrichment by separation in a discontinuous density gradient and then ultracentrifuged in a Beckman Coulter Optima L-100K Ultracentrifuge equipped with a fixed-angle rotor Type 50.2 Ti (Beckman Coulter, Brea, CA, USA). Lysosome pellets were solubilized in a lysis buffer containing 7 M urea, 2 M thiourea, 4% CHAPS and protease inhibitors. Protein concentration was determined using the Bio-Rad protein assay (Bio-Rad Laboratories, Inc., Hercules, CA, USA). Both the degree of lysosomal enrichment and cross-contamination level with other cellular compartments were evaluated in control HEK293T cell line by Western blot (Supplementary Figure S1). Cells were probed with specific antibodies for lysosomal compartment (CTSD and LAMP2 like soluble and membrane lysosomal marker, respectively), mitochondria (SDHA and Core2), and cytosol (GAPDH).

### 2.3. Proteomic Analysis

Lysosomal fractions obtained from either HEK 293T cell lysates or mice cerebral cortices were processed and used for DIA-HDMS^E^ (data independent acquisition-high-definition ion-mobility enabled tandem mass spectrometry), as described elsewhere [11]. Database searches were carried out against human (release 2017_48461 entries) or *Mus musculus* (release 2017_16869 entries) UniProtKB/SwissProt reviewed databases. A label-free protein quantitation method was applied for post-processing data analysis using precursor ion intensity data and standardized expression profiles. The proteomics data were submitted to MassIVE (accession number MSV000088517).

### 2.4. Development of a Scoring System for Lysosomal Proteins and Data Confidence

Several lysosomal candidate proteins have been surveyed in multiple proteomic studies involving different sources of material, purification approaches, and compartmental assessment methodology. Distinguishing true organelle constituents from contaminants is crucial in subcellular proteomics, with particular consideration for lysosomes as sites of degradation for many intracellular and extracellular proteins. To overcome this issue, we conceived a hierarchical confidence scoring system for lysosomal proteins, combining lysosomal annotations from different databases and studies.

**Figure 1:**
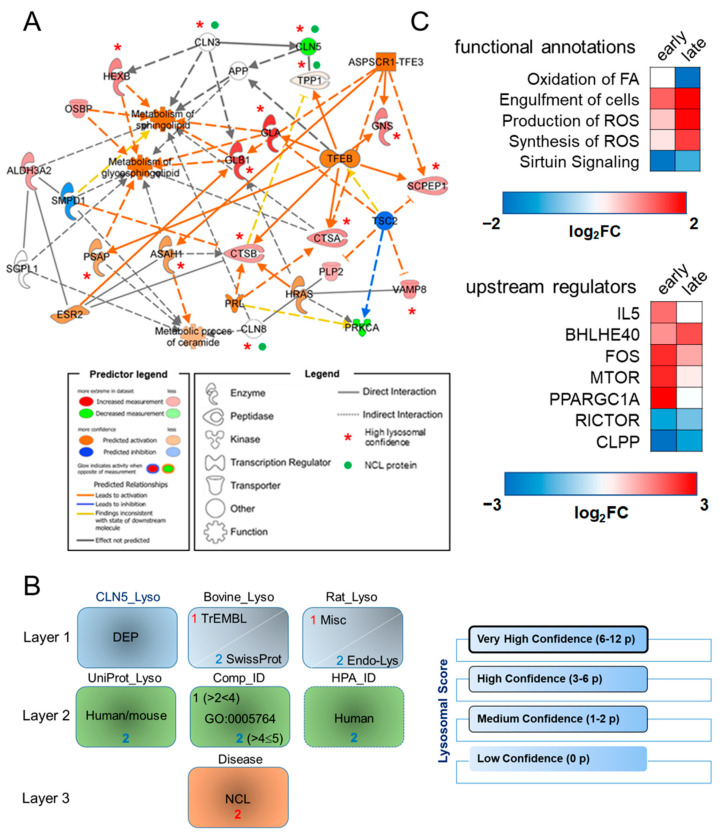
Lysosomal bioinformatics survey in cellular and murine models of CLN5 disease. (**A**) Functional association network encompassing differentially expressed proteins assigned with high and very-high-confidence lysosomal scores (red asterisks), concomitant diseases and functions and predicted upstream regulators in the CLN5 KO cellular model. The CLN5 knock- out model is linked to alterations in sphingolipid transport. NCL proteins (marked by green dots) and APP (Amyloid b precursor protein) were added as connecting nodes, linking CLN5p to other NCL disorders and Alzheimer’s disease. (**B**) Development of a lysosomal scoring system. DEP identified in different CLN5 models were queried in different MS-based lysosomal datasets (rat and bovine; confidence layer 1, TrEMBL- 1 point; UniProtKB/SwissProt- 2 points). Lysosomal confidence in layer 2 was given if the protein was identified in human/mouse UniProtKB dataset (2 points), Compartments database (GO:0005764, lysosome; 1–2 points) and Human Protein Atlas, HPA (2 points). Layer 3 (NCL score, 2 points) was derived from lysosomal datasets described in Schmidtke, C., 2019 (CLN3), Tuermer, A., 2021 (CLN6), Danyukova, T., 2018 (CLN7), Klein, Z.A., 2017 (CLN11). In combination, this scoring system resulted in various levels of lysosomal confidence (right panel). The full description is given in the Materials and Methods sections and Supplemental Data. (**C**) Top meaningful functional annotations and upstream regulators reported along the disease progression are indicated in the *Cln5*^−/−^ mouse brain lysosomes. Early—early phase; late—late phase of disease status. The activation state for each biological function or upstream regulator is indicated by the respective activation z-score.

Differentially expressed proteins (DEP) were identified based on the number of unique peptides used for label-free quantitation (≥2), at the FDR < 0.01 and the fold change (FC) from averaged, normalized protein intensities |≥1.3| for lysosomal mouse brains datasets and |≥1.5| for HEK 293T cell lines, utilizing *p* ≤ 0.05 by ANOVA in all comparisons. Protein identifiers (IDs) attained in HDMS^E^ analysis were further filtered for lysosomal localization and ranked based on combined annotations from various lysosomal databases. Layer 1 of confidence included SWATH™ data from bovine [19] (TrEMBL, 1 point and SwissProt, 2 points), rat lysosomal proteome (miscellaneous (Misc; vesicles, granules, and multiple localizations; 1 point), and endo-lysosomes (EL, 2 points) [20]. Layer 2 comprised curated data of human and mouse lysosomal proteins (hLGDB and mLGDB; 2 points) [21,22], and the reviewed sections of UniProt Knowledgebase with a keyword lysosome [23], Compartments database (GO:0005764, lysosome; confidence score >2 and <4 was awarded with 1 point while those with a confidence score ≥4 and ≤5, were awarded 2 points; https://compartments.jensenlab.org/; accessed on 26 April 2022) and Human Protein Atlas, HPA (lysosomal localization; https://www.proteinatlas.org/; 2 points, accessed on 26 April 2022). NCL score (layer 3, 2 points) was obtained matching our lysosomal datasets with those from experimental models of other NCL forms [12,13,14,15]. The final score is represented by various levels of lysosomal confidence encompassing very-high-confidence protein data with 6–14 points (Lyso vHC dataset), high confidence with 3–6 points (Lyso HC set), medium confidence scoring 1–2 points (Lyso MC dataset) and of low confidence scoring 0 points. Criteria employed in the scoring system are summarized in Supplementary Table S1 and presented in Figure 1B. IDs with a score below 1 were discarded.

### 2.5. Detection and Quantification of Intracellular Lipids by Fluorescence Microscopy

Cells were incubated overnight in growth media supplemented with excess lipids (400 µM oleic acid complexed to albumin, OA/BSA) to enhance lipid droplet formation and then allowed to rescue for 2 or 24 h in regular medium, in the presence or absence of selected drugs. Images were acquired using a Nikon Ti2-E inverted microscope equipped with ViCo structured light system, using a ×60 Plan Apocr λ (NA = 1.40) oil immersion objective and an FITC filter set. For data quantification, we employed an unbiased method suitable for immunofluorescence staining. In particular, images in raw green channel (Bodipy 493/503) were processed by applying local thresholding and then analyzed by the “analyze particles” command of ImageJ software [24]. To select and to count the droplet-like structures in each field, we generated a count mask, by setting the parameters of size and circularity. Objects with circularity >0.6 and area greater than 3 µm^2^ were counted and normalized to the number of cells defined upon Hoechst staining (Supplementary Figure S2).

### 2.6. Cell Treatment

Both SH-SY5Y cell lines and primary fibroblasts from CLN5 patients and control donors were treated with miglustat (N-butyldeoxynojirimycin, Santa Cruz Biotechnology, Dallas, TX, USA) or trehalose (D-(+)-Trehalose dihydrate, Sigma Aldrich, St. Louis, MO, USA). Chemical substances were dissolved in regular cell culture medium and filtered through a sterile filter membrane with 0.22 μm pore size before use. Cultured skin fibroblasts and SH-SY5Y cells were then incubated with the respective drug at 37 °C in 5% CO_2_. Concentrations and times used were as follows: 100 μM miglustat for 72 h and 200 mM trehalose for 72 h. To enhance lipid droplet formation, cells were incubated overnight in growth media supplemented with an excess of lipids (400 µM oleic acid complexed to albumin, OA/BSA). Rescue experiments were performed following drug treatment by incubating the cells for either 2 or 24 h in regular medium or in the presence/absence of selected drugs. The used drug concentrations are based on the results from previous experiments and literature findings [25,26,27,28].

### 2.7. Functional Studies on Zebrafish

Concentrations of morpholino were carefully titrated to avoid nonspecific binding effects and a scrambled control morpholino was used at similar concentrations to assess specificity to *cln5*. After titration, 5 ng of splice morpholino against *cln5* was used in all experiments. Rescue experiments were performed through co-injection of 50 pg of control, *cln5* WT cRNA with splice morpholino at the same concentration used for the knockdown experiments. Each experiment was repeated at least three times if not otherwise stated. Rescue experiments were also analyzed at 48 hpf through touch-evoked escape response [29]. Locomotion behavior was acquired using Danio Vision at 120 hpf and data analyzed using EthoVision software (Noldus, Wageningen, The Netherlands). After 30 min of adaptation, larval locomotion was analyzed for 30 min. Locomotor activity in response to light–dark conditions, also known as visual motor response (VMR), was analyzed using the Danio Vision system and the locomotion was analyzed through 4 cycles of alternating light and dark periods of 10 min each. For each experiment, at least three independent assays were performed. The transgenic *Tg(Neurod1-GcAMP6f)* line was used to inspect brain/head morphology in 72 hpf embryos after morpholino injection. To quantify possible eye abnormalities, we measured the eye size using Danioscope software (Noldus). Electrophysiological forebrain recordings were performed in 120 hpf larval zebrafish, as described [30]. The whole-mount immunohistochemistry staining was performed as reported [29] utilizing anti-ATP synthase subunit C (SCMAS) antibody (1:100; Abcam, Cambridge, UK). LysoTracker Green DND-26 (L7528, Thermo Fisher Scientific, Waltham, MA, USA) was used to stain lysosomes and other acidic organelles in live zebrafish larvae. Zebrafish larvae at 5 dpf were incubated with the dye for 1 h in the dark. Following the staining, larvae were rinsed 3 times with fresh egg water. All images were acquired using the stereomicroscope Leica M205FA (Leica, Wetzlar, Germany), and fluorescence analysis was performed using Image-J software v.1.46 calculating the fluorescent intensity in the region of interest (ROI).

## 3. Results

### 3.1. Proteomic Analysis and Bioinformatic Categorization

The whole datasets reporting the differentially expressed proteins (DEP) in CLN5 disease models are presented in Supplementary Tables S2–S4.

Mass spectrometry analysis of purified lysosomal fractions from cells with *CLN5* knockout allowed the quantification of 1170 proteins. Upon statistical filtering, 183 DEPs were identified, of which 129 (~70% of the total) were above the set lysosomal confidence (24, 18, and 87 DEPs assigned with the “Very High”, “High” and “Medium Lysosomal Confidence”, respectively). Bioinformatic categorization of lysosomal DEP was carried out using Ingenuity Pathway Analysis (IPA) software platform (QIAGEN, Hilden, Germany), by employing the ratios of normalized relative abundance intensities of the reported DEP, as described [31]. A statistical prediction, amenable to portray the activation state of specific dysregulated pathways, demonstrated an overload of *sphingolipids*, *glycosphingolipid,* and *ceramide metabolism,* with possible implications in sphingolipid transport, *engulfment of cells,* and affected *neuritogenesis*. ASPSCR1-TFE3, TFEB, HRAS, PRKCA, and TSC2 were proposed as upstream regulators with predicted activation (as judged by positive z-score >2) that can explain the observed protein abundance changes. The parameters of activation (z-scores, *p*-values, and the molecules involved in the identified *Disease and Function* annotation and *Upstream regulators*) are reported in Supplementary Tables S5 and S6. The links among lysosomal DEP and their associated *Diseases and Functions* and *Upstream regulators* are displayed in the functional interaction map presented in Figure 1A. In a subsequent step, different NCL proteins (TPP1/CLN2, CLN3 and CLN8) were added as bridging nodes to the network of functional relationships encompassing CLN5 knockout, thereby reinforcing the hypothesis of a common basis of pathogenicity among various NCL forms. Proteomic profiling of lysosomes derived from brain cortex of *Cln5*^−/−^ mice, identified 1615 and 1610 proteins at the early (presymptomatic) and late (symptomatic) disease stages, yielding 362 and 180 DEPs upon bioinformatic filtering, respectively.

Comparisons between disease stages were performed to highlight the most significant pathways and upstream regulators related to disease progression. We identified several annotations related to dysregulated neuronal development and engulfment of cells (Supplementary Table S7). Stringent scoring for lysosomal confidence further revealed 123 DEPs at the early stage (~34% of the total, comprising 15, 16, and 92 DEPs with the “Very High”, “High” and “Medium Lysosomal Confidence”, respectively) and 63 DEPs from the late stage (~34% of the total, with 7, 6, and 48 DEPs with the “Very High”, “High” and “Medium Lysosomal Confidence”, respectively, Figure 1C). Analysis of these lysosomal datasets highlighted metabolic pathways related to the fatty acid oxidation as a source of the increased ROS production and decreased Sirtuin signaling, modulating oxidative stress and protecting the cells against ROS. Among upstream regulators, *Rapamycin-insensitive companion of mTOR* (RICTOR) showed increased dysregulation, linking proteomic changes occurring across disease stages (Supplementary Table S8). To identify the most relevant biomarker candidates within our experimental data, we performed IPA-assisted biomarker filtering. We selected the parameters that were most relevant to biomarker discovery in LSDs, by filtering the data for species (mouse), node types (enzymes), disease category (hereditary, developmental, neurological, metabolic, inflammatory, skeletal muscle diseases), allowing us to gather disease-relevant candidate biomarkers. Moreover, we set a threshold of detectability, selecting for its detection in at least one compartment, i.e., the detection in blood and serum/plasma or in the brain. Utilizing this strategy, we pinpointed a list of potential molecular biomarker candidates, for the early and late disease stage (Supplementary Table S9). Among these, PARK7 (Parkinson disease protein 7) and SNAP25 (Synaptosome associated protein 25), known to be involved in the pathology of CLN1 and CLN4 diseases [32,33], were reported as transversal biomarkers with consistent levels of dysregulation across the disease progression. Moreover, several enzymes involved in glycolysis/gluconeogenesis were also highlighted, particularly at the presymptomatic stage, with a visible trend for an upregulation.

### 3.2. Validation of Selected DEP

**Figure 2:**
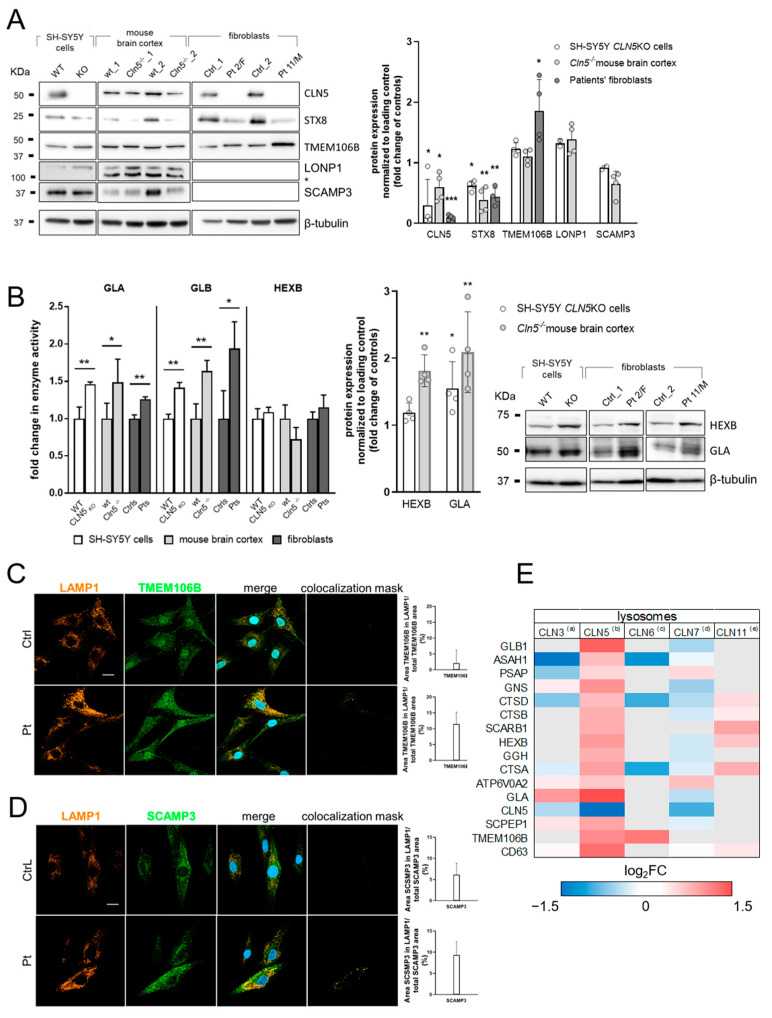
Cross-validation of lysosomal proteomic profiling data. (**A**) Differentially expressed proteins identified in lysosomal proteomic profiling, and assigned with very-high (CLN5, TMEM106B), high (STX8, SCAMP3) or medium (LONP1) lysosomal confidence scores, were selected to assess their differential abundance by Western blot in whole cell lysates and patients’ fibroblasts corroborating the results of proteomic profiling. WT, wild type (wt); KO, CLN5 knockout SH-SY5Y cells; *Cln5*^−/−^, Cln5 knockout mouse brain cortex; Ctrl, cultured skin fibroblasts derived from healthy subjects; Pt, CLN5 cultured skin fibroblasts from CLN5 index cases. (**B**) Assessment of protein abundance levels by Western blotting and corresponding enzymatic activity of selected differentially abundant lysosomal enzymes related to sphingolipid metabolism. In SH-SY5Y cells, mouse brain cortex and patients’ fibroblasts, the results well correlated with the upregulation in abundance seen in the proteomic profiling. Representative confocal images of selected proteins with very-high (**C**) and high (**D**) lysosomal confidence and their accumulation within the lysosome. Colocalization analysis indicates an increased shared area between lysosomal compartment (LAMP1 staining) and the protein of interest (TMEM106B and SCAMP3). Data were calculated and normalized to the total intensity area given by the green channel. Histogram refers to the average colocalized area ± SD calculated on five different fields. Scale bars: 10 µm. (**E**) Heat map reporting differentially expressed lysosomal proteins identified in this study. The map also shows protein abundance derived by other lysosomal omics studies in NCL models. (a) Schmidtke, C., 2019 (CLN3), (b) this study (CLN5), (c) Tuermer, A., 2021 (CLN6), (d) Danyukova, T., 2018 (CLN7), (e) Klein, Z.A., 2017 (CLN11). Statistical difference between groups was assessed by two-tailed unpaired *t*-test. *** *p* ≤ 0.001, ** *p* < 0.01, * *p* ≤ 0.05.

Among lysosomal DEP detected in the KO cell model, we selected enzymes assigned with ”Very-High” lysosomal confidence related to sphingolipid metabolism (alpha-galactosidase A, GLA and beta-hexosaminidase subunit beta, HEXB), endosomal maturation (syntaxin 8, STX8), and stabilization of the levels of lysosomal enzymes (transmembrane protein 106B, TMEM106B), and two out of five common DEP between cell and mouse models (Lon peptidase 1, mitochondrial, LONP1 and secretory carrier membrane protein 3, SCAMP3) (Supplementary Table S2). Protein abundance changes, including CLN5p, were assessed by Western blot in cell lysates, mouse brain cortex, and patients’ fibroblasts, corroborating the results of proteomic profiling (Figure 2A). To normalize the differences in protein abundance in each lane, we used a load control (β-tubulin). Moreover, we tested for the hydrolase activity of differentially expressed lysosomal enzymes involved in sphingolipid metabolism and observed a significant increase in GLA and GLB (but not HEXB) enzyme activities, supporting, at least in part, the differential increase in abundance of these enzymes seen in the proteomic profiles. GLA and HEXB levels were also assayed by Western blot, correlating protein abundance with cellular proteomic profile (Figure 2B). To validate the developed lysosomal scoring system, we performed a colocalization analysis of selected proteins with ”Very-High” (TMEM106B, Figure 2C) and “High” (SCAMP3, Figure 2D) lysosomal confidence within the lysosomal compartment (LAMP1 marker). In line with the results of lysosomal proteomic profiling, we detected an increased colocalization of target proteins within lysosomes from CLN5 patients. A sizable subset of lysosomal proteins with “High” and “Very-High Lysosomal Confidence2 (lysosomal DEP in cellular CLN5 model) has been reported in other NCL-related lysosomal datasets (Figure 2E), strongly reinforcing the hypothesis of common molecular theme(s) across different NCL forms and suggesting the need for further studies to assess their role as possible disease markers.

### 3.3. Characterization of the Zebrafish cln5 Knockdown Model

Full characterization of the *cln5* knockdown model in zebrafish, using splicing morpholino, is reported in Supplementary Figure S3 and Supplementary Table S10.

**Figure 3:**
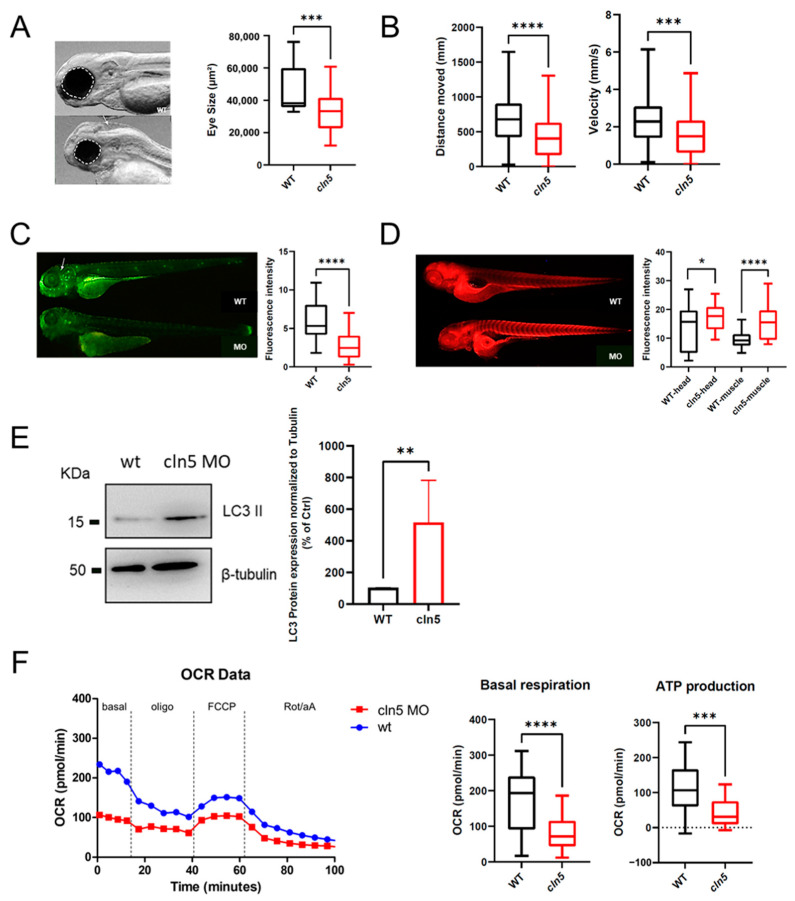
Characterization of *cln5* morphant vertebrate model. Zebrafish *cln5* morphant model recapitulates most of the pathological features observed in the human disease. (**A**) Eye size measurement showing a reduced ocular surface in *cln5* morphants compared to control siblings. *n* = 30 embryos per condition (**B**) Locomotion analysis of *cln5* morphants (*cln5* MO) and control siblings (wt). *n* = 81 embryos per condition. (**C**) Lysotracker staining and fluorescence analysis in morphant (cln5) and unaffected sibling embryos (wt). *n* = 45 embryos per condition. (**D**) Immunofluorescence analysis of SCMAS in muscle and brain of morphant (cln5) and control (wt). *n* = 52 embryos per condition. (**E**) LC3 II Western blotting in morphants (cln5) and control siblings (ctrl). *n* = 52 embryos per condition. (**F**) Micro-oxygraphy track showing a reduced OCR in morphants (*cln5*) compared to control siblings (wt). Basal registration without compound treatment was followed by the injection sequence of complex V inhibitor Oligo (oligomycin); FCCP to boost to the maximal respiration; combination of antimycin A and rotenone for a complete block of mitochondrial respiration. *n* = 28 for morphants, *n* = 30 for control siblings. Significant differences in basal OCR and ATP production are shown. Statistical difference between groups was assessed by Mann–Whitney test. **** *p* ≤ 0.0001, *** *p* < 0.001, ** *p* < 0.01, * *p* < 0.05.

The zebrafish *cln5* morpholino model recapitulates most of the pathological features observed in CLN5 disease. A reduced ocular surface was measured in the *cln5* morphants compared to control embryos (Figure 3A), whereas a locomotor impairment of the *cln5* morphants was displayed both in terms of velocity and distance covered (Figure 3B). Immunofluorescence analysis revealed the typical accumulation of the marker subunit c of mitochondrial ATP synthase (SCMAS), as well as an increased lysosomal activity, further demonstrated by enhanced levels of the lipidated form of LC3 (LC3-II), usually linked to the mature stage of autophagosomes (Figure 3C–E). Micro-oxygraphy analysis of cellular respiration pinpointed a reduced basal oxygen consumption rate (OCR) and ATP production in *cln5* morphants compared to control siblings (Figure 3F), corroborating the results of the impaired bioenergetics shown in patients’ fibroblasts [11].

### 3.4. In-Vitro Modulation of ROS Overproduction

**Figure 4:**
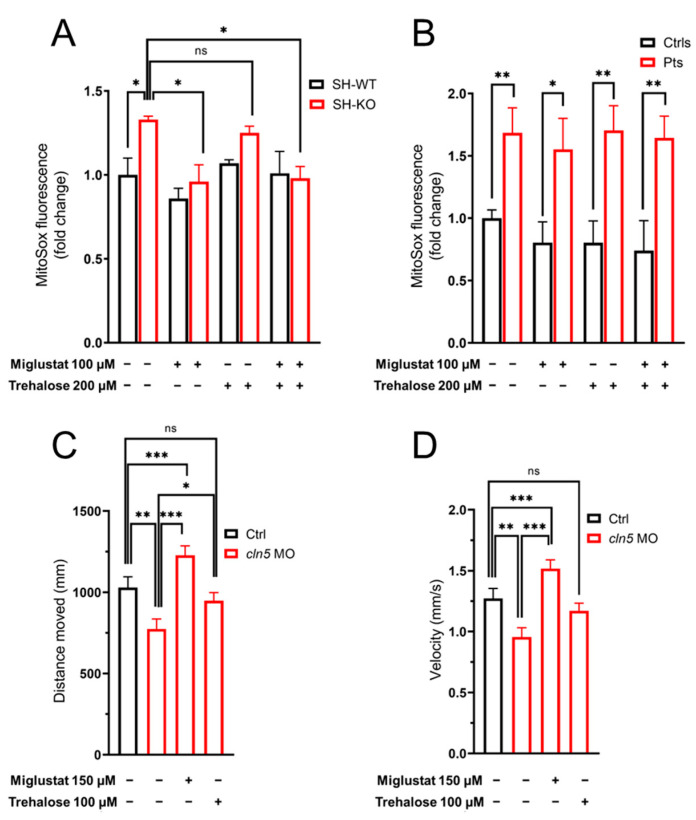
Functional in-vitro and in-vivo studies in CLN5 models. Mitochondrial ROS levels by MitoSOX staining intensity in the neuronal-like model, SH-SY5Y cells (**A**) and patients’ fibroblasts (**B**) were assessed in the presence/absence of two 340 drugs, namely, miglustat and trehalose. Data refer to two technical replicates form two independent experiments. Two-tailed unpaired *t*-test. *** *p* ≤ 0.001, ** *p* < 0.01, * *p* ≤ 0.05. (**C**,**D**) Effect of misglustat and trehalose on locomotor impairment in *cln5* zebrafish morphants. The picture shows locomotor performances (distance and velocity) of wild-type (Ctrl), untreated *cln5* morphants (*cln5* MO) and morphant larvae treated with misglustat and trehalose. Tracking experiments were performed using *n* = 273 wt, *n* = 190 cln5MO, *n* = 145 miglustat-treated *cln5* MO embryos, and *n* = 134 trehalose-treated cln5MO embryos.

To explore the possible consequences of disturbances in lipid metabolism, autophagy, and mitochondrial dysfunction in the pathogenesis of CLN5 disease, we evaluated the levels of oxidative stress in both the cell model and patients’ fibroblasts. We measured the oxidation of MitoSOX Red reagent by assessing the amount of ROS generated in mitochondria. Fluorescence measurements showed a significant increase in susceptibility to oxidative stress in SH-SY5Y cells (SH-model) and in all tested patients (Figure 4A,B). Treatments with both miglustat and the combination of miglustat + trehalose reduced the mitochondrial reactive oxygen spices in the SH model. However, no significant changes were observed in skin fibroblasts from three index cases.

### 3.5. Modulation of Locomotor Phenotype in the Zebrafish Model

In the *cln5* morphant model we tested the possibility to ameliorate the motor performances of mutant embryos using the aforementioned compounds. We observed that miglustat and trehalose could rescue locomotor impairment in a treated *cln5* morphant (Figure 4C,D), corroborating in vivo the efficacy of these xenobiotics in alleviating some of the disease symptoms observed in vitro.

### 3.6. Modulation of Lipid Overproduction

**Figure 5:**
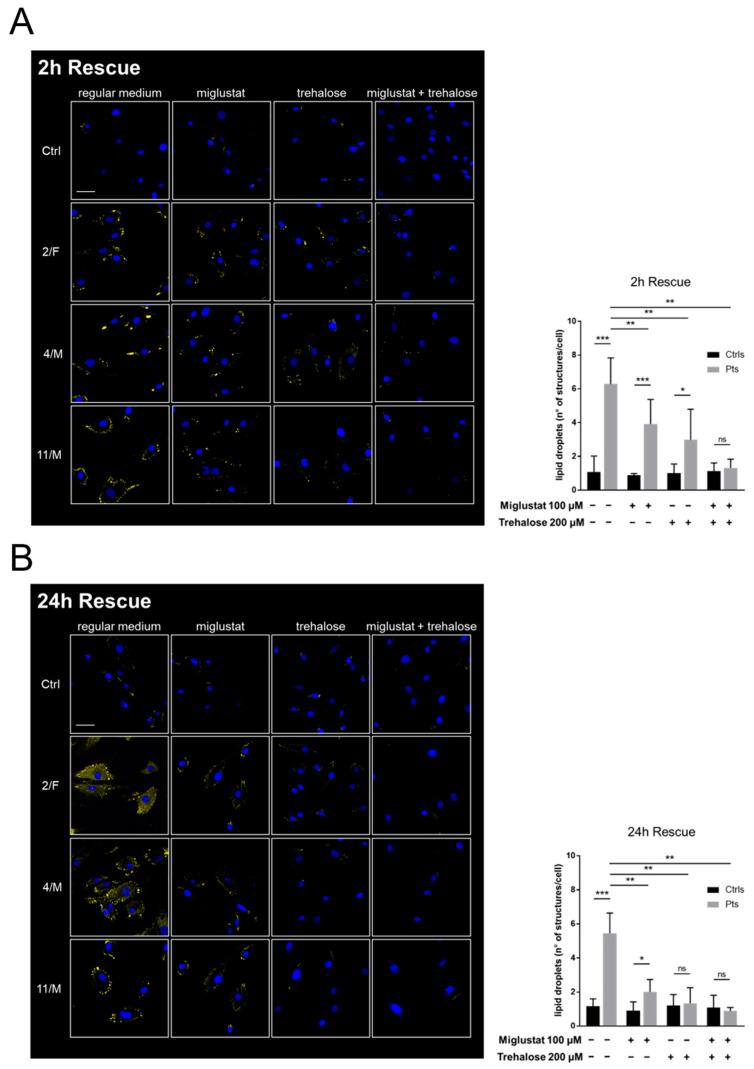
Quantification of lipid storage in patients’ cells and modulation of lipid overload by compound treatments. Cells were incubated overnight with lipid excess and the number of lipid droplet-like structures quantified by BODIPY 493/503 after 2 h (**A**) and 24 h (**B**) of rescue with regular medium in the presence/absence of drug treatments. Representative images for lipid detection are shown. Data are presented as means, *n* = 50 cells from three different CLN5 patients (Pts) and three healthy controls (Ctrls). Two-tailed unpaired *t*-test. *** *p* ≤ 0.001, ** *p* < 0.01, * *p* ≤ 0.05.

Lipid droplets (LDs) are ubiquitous, dynamic organelles and function as a storage depot for neutral lipids. Lysosomes are involved in the metabolism of lipid droplets and an accumulation of LDs has been related with lysosomal and autophagy alterations [34]. In the basal condition (ON incubation in growth media supplemented with excess of lipids to enhance LDs formation), all the three analyzed CLN5 patients cell lines showed a similar increase (~5 and 6-fold) in the number of LD-like structures, following 2 and 24 h of rescue in regular medium. After 2 h of rescue following miglustat or trehalose treatment, we observed a significant decrease for about 40 and 50%, respectively, in the number of highlighted structures, in contrast to the basal levels seen in the controls. The combination of drugs showed a sharp decrease in the number and dimensions of the structures already after 2 h of rescue in regular medium (Figure 5A). Following 24 h of rescue, there were no significant changes in the number of LDs as compared to 2 h, whereas we observed a decrease in LDs by >60% once cells were treated with miglustat and compared to those untreated. Similarly, 24 h after trehalose rescue, as well as the combination of miglustat and trehalose, induced a sharp fall in the number of LDs, bringing it down to control levels (Figure 5B).

## 4. Discussion

Omics approaches have revolutionized research in the field of rare diseases with the generation of multilayer information, in spite of a limited set of patients’ material and not always precise genotype–phenotype correlations. In this study, we implemented an organelle-focused, label-free quantitative proteomics approach to yield new information on disease pathogenesis, biomarkers, and potential therapeutic targets in a rare, devastating form of childhood neurodegeneration. The CLN5 protein has been implicated in mitochondrial bioenergetics, mitophagy, lipid metabolism, lysosome receptor sorting, myelination, and sphingolipid transport [10,11,35,36]. However, the precise mechanisms underlying CLN5 disease are yet to be revealed. In previous work [11], we utilized a functional proteomics approach focused on a specific organelle to define a new mitochondrial role of CLN5 protein. In this study, we applied compartmental proteomics and focused on the CLN5p function at the lysosomal level.

Efficient enrichment of lysosomes from whole cells and tissue extracts was gained by an optimized density-based separation method. A Western blot analysis of control cells was used as a quality checkup of the fractions resulting from the centrifugation process (Supplementary Figure S1). Using the filtering system developed in this study (see *Development of a scoring system for lysosomal proteins and data confidence*), we demonstrated that almost 70% of proteins with differential abundance in the CLN5 cellular model were assigned with a stringent lysosomal confidence score, whereas the percentage of DEP from the mouse brain cortex to which a lysosomal score could be assigned amounted only to one-third. For this reason, in further analyses, we scrutinized only those *Cln5*^−/−^ brain-derived DEP, to which a lysosomal score could be assigned.

Several disease models, including KO cells, *Cln5*^−/−^ mice, and *cln5* morphant zebrafish, were analyzed for precise scrutiny/validation of affected pathways and potentially offers new functional readout related to the lysosomal impairment in NCL. Categorization of DEPs indicated an overload of lipids and increased susceptibility to oxidative stress, as the most relevant events in CLN5 disease pathology. Sphingolipids are abundant in nervous tissue and regulate several cell functions. They accumulate in tissues, including the brain, upon oxidative stress, while levels of enzymes related to sphingolipids metabolism are elevated, resulting in enhanced generation of ROS [37]. Altered lipid metabolism functionally links with the activation of autophagy or mitophagy, as previously inferred by our study on mitochondrial involvement in CLN5 disease [11]. Similar processes were also demonstrated in different NCL disease models, including *CLN5* KO neuronal-like cells [11] and in CLN5 patients’ [38]. Analyses in the brain cortex of *Cln5*^−/−^ mice [39] indicated disturbances in neuritogenesis and the engulfment of cells, whereas the categorization of a specific lysosomal dataset resulting from a more stringent filtering system highlighted signaling pathways related to the oxidative stress mechanism, while maintaining the engulfment of cells as functional annotation. Furthermore, RICTOR was pinpointed as an upstream regulator behind the observed differential protein abundance. Pathways related to RICTOR have also been implicated in the maintenance of neuronal stability and modification of a phenotype in in-vivo models of NCLs [40,41], and more recently, as a top upstream regulator of proteomic dysregulation present across the majority of NCLs [42]. Analysis of mouse model advances the identification of lysosomal-related molecular signatures in terms of both disease status and progression. Filtering analysis indicated common biomarkers between presymptomatic and symptomatic stages, including PARK7, a neuroprotective protein against neuronal injury, encoded by a gene involved in early-onset Parkinson’s disease, reinforcing the intriguing item of a possible relationship with NCLs and more common forms of neurodegeneration.

Among the upstream regulators in the *CLN5* KO cellular model, we identified *TFEB*, the master gene regulator of lysosomal biosynthesis, *PPARGC1A*, the master gene regulator of mitochondrial biogenesis, implicated also in the process of neuronal death, and INSR, involved in neuroprotection. Further strengthening the results of our lysosomal proteomic profiling and functional validation experiments, we detected the involvement of ganglioside metabolic pathways in CLN5 disease. Elevated protein levels related to increased enzyme activities of GLA and GLB were detected across CLN5 disease models. Increased abundance of GLA, the protein with the highest expression fold change in the KO model, was detected across the studied disease models and further functionally validated by the respective high levels of enzyme activities (Supplementary Figure S1B). Such reductions in the GLA protein levels have been previously demonstrated in *Cln10* knockout mice [43], *CLN5*-deficient human fibroblasts [44], and *CLN8* knockout cells [36]. Products of sphingolipids metabolism are associated with oxidative stress and act as second messengers to increase oxidant production [45,46]. Moreover, increased ROS activity can stimulate sphingolipids turnover [47]. Furthermore, autophagy (either macro- or mitophagy) and dysregulation of lysosomal clearance have been implicated in several forms of NCL [11,48], and increasing evidence points out that mitochondrial ROS represent the upstream modulators of autophagy. Our proteomic profiling indicated several autophagy-related proteins, including STX8 and TMEM106B, with differential abundance validated by Western blot. STX8 is involved in endosome maturation and provides an inhibitory activity for Cl^−^ ion channels, whereas the strong upregulation of TMEM106B is related to the dysregulation of lysosomal enzyme levels, since loss of expression of the latter was demonstrated in *Grn*^−/−^ mice as their stabilizer. Interestingly, dysregulation of bioenergetics and the endosomal–lysosomal system is increasingly gaining more attention as a putative culprit of pathology in several forms of NCL disease [11,48]. Mitochondrial ROS may represent upstream modulators of autophagy, as suggested for different models of dementia [49,50].

We identified five dysregulated proteins across all the models (RAB6A, ERP29, SCAMP3, TNIK, and LONP1). Among these, LONP1 has been recently associated with dysfunction in energy metabolism that promotes severe neurologic impairment and neurodegeneration [51]. SCAMP3 is involved in recycling systems, including the trans-Golgi network, early sorting, and recycling endosomes [52]. By utilizing the molecule activity prediction (IPA), the directional consequences of downstream pathway “lipid metabolism” downregulation and “metabolic process of ROS” activation were linked to the measured altered abundance of these common DEP and putative upstream regulators (Supplementary Figure S4).

Taken together, these results reinforce our understanding of an interplay between lipid metabolism, autophagy, and oxidative stress, thereby leading to the modulation of cellular homeostasis, cell survival, and death. The network of dysregulated lysosomal proteins in the *CLN5* KO model links it further to other NCL proteins (TPP1/CLN2, CLN3, and CLN8) and cathepsins. Such connectivity promotes crosstalk among various NCL forms, supporting CLN5 functional connection to PPT1, TPP1, CLN3, CLN6, and CLN8, seen in in-vitro experiments [6,7] and other NCL proteins [4]. Moreover, differentially expressed lysosomal proteins identified in this study have been found dysregulated in their abundance in other lysosomal omics studies utilizing experimental models from other NCL forms [12,13,14,15]. Altogether, this strengthens a scenario of a common pathological pathway(s) amenable for future targeting in NCL pharmacological studies.

In this work, we focused on the analysis of lipid excess and increase in oxidative stress and introduced a novel, promising readout to evaluate the possibility of a pharmacological modulation in CLN5 disease. The possible implication of the ganglioside signaling pathway offers potentialities to investigate FDA-approved drugs promoting autophagy, with increased interest in NCL research; a proprietary combination of miglustat [53] and trehalose, has already been proposed for the treatment of CLN3 disease (BBDF-101). This combination will soon be clinically trialed in Batten disease (https://beyondbatten.org/, accessed on 2 May 2022). Our results indicate that the use of miglustat appears to be more effective than trehalose, especially in the modulation of ROS overproduction, allowing a rescue to the control levels, although only in a CLN5 KO cell model. We further confirmed these findings by rescuing locomotor activity in *cln5* morphants upon combined treatment. However, miglustat therapy did not appear to alleviate the oxidative stress levels in cells from CLN5 index cases, suggesting that a combination therapy with antioxidants may likely provide additional benefits. This hypothesis is under investigation in the zebrafish model [54]. Both drugs tested in our study seem to be involved in the modulation of lipid generation and, when in combination, they provide additive effects, as observed by others in *Cln3*^−/−^ mice [41]. It is tempting to hypothesize the possibility to expand the efficacy of trehalose and its combination with miglustat in other forms of NCL, including CLN5, characterized by an activation of the autophagy–lysosome pathway.

In summary, the current study highlights the role of the CLN5 protein in sphingolipid metabolism and in the positive modulation of oxidative stress, offering new information on disease pathogenesis, biomarkers, and potential therapeutic targets. We also evaluated the possibility of a pharmacological modulation not only in vitro but also in an ad-hoc engineered *cln5* zebrafish model. Our findings might have a direct translational value if one considers the promising readouts to be implemented into studies on NCL patients’ material, to monitor their disease progression.

## Data Availability

All data generated or analyzed during this study are included in this published article and its Supplementary Information Files. Mass spectrometry data were deposited in the MassIVE database under accession number MSV000088517.

## Supplementary Materials

The following are available online at https://www.mdpi.com/article/10.3390/cells11111840/s1. Supplementary Figure S1: Monitoring the degree of enrichment and contamination level in mitochondrial and lysosomal preparations. Supplementary Figure S2: Unbiased method for detection and quantification of intracellular lipids. Supplementary Figure S3: Analysis of zebrafish CLN5 ortholog. Supplementary Figure S4: Lysosomal DEP identified across cellular and murine models. Supplementary Table S1: Scoring system for lysosomal proteins. Supplementary Table S2: Lysosomal assignment and DEP in the cellular *CLN5* KO model. Supplementary Table S3: Lysosomal DEP in the presymptomatic *Cln5*^−/−^ mouse model. Supplementary Table S4: Lysosomal DEP in the symptomatic *Cln5*^−/−^ mouse model. Supplementary Table S5: Disease and function annotation in the *CLN5* KO model. Supplementary Table S6: Upstream regulators in the *CLN5* KO model. Supplementary Table S7: Comparative analysis of disease and function annotations in *Cln5*^−/−^ mice. Supplementary Table S8: Comparative analysis of upstream regulators in *Cln5*^−/−^ mice. Supplementary Table S9: Biomarker filter analysis. Supplementary Table S10: Target sequences and primers used to create morpholino *cln5* zebrafish model. References [55,56,57,58,59,60,61,62,63,64,65] are cited in the supplementary materials and methods.
