# Supplementary material for: Lysosomal Proteomics Links Disturbances in Lipid Homeostasis and Sphingolipid Metabolism to CLN5 Disease
Source: Cells. 2022 Jun 4;11(11):1840. doi: 10.3390/cells11111840 (PMC9180748; doi:10.3390/cells11111840)
Supplement: Supplementary file 1 [file cells-11-01840-s001.zip › cells-1727854-supplementary.pdf]

Supplementary materials and methods

In vitro and _in vivo_ disease models

HEK 293T cell line (293T ATCC® CRL-3216™), derived CRISPR KO (hereafter termed KO) and empty-vector px459 expressing cells (hereafter termed Ctrl) were cultured in DMEM containing 10% FBS, 4.5 g/L glucose and 1% penicillin/streptomycin, supplemented with l-glutamine to achieve a final concentration of 6 mM. Neuroblastoma cell line (SH-SY5Y ATCC® CRL-2266) and derived KO and Ctrl clones were cultured in MEM/F12 1:1 with 10% FBS, 2 mM l-glutamine, 1% penicillin/streptomycin. A detailed description of the protocol for genome engineering of HEK 293T and SH-SY5Y KO cell lines by the CISPR/Cas9 system, together with their proper characterization has been previously published [11]. Primary cultured skin fibroblasts from CLN5 index cases harboring different mutations in the *CLN5* gene have been described elsewhere [38] and were already collected in our Center, whereas control fibroblasts were collected according to standard procedures from diagnostic skin biopsies following a signed informed consent form that authorized research uses in accordance with our CEPR (Tuscany Region Ethic committee). All primary cell lines were grown at 37 °C with 5% CO_2 in Dulbecco's modified Eagle's medium (DMEM), containing 10% fetal bovine serum (FBS), 4.5 g/L glucose and 1% antibiotics/antimycotics. The *Cln5* mouse model of late infantile Finnish variant of NCL was originally generated at the University of Helsinki [39], by disrupting exon 3 of the murine *Cln5* gene, resulting in a premature stop codon in exon 4. Both male and female mice at pre-symptomatic (3 months) and symptomatic stages (9 months) were used. Age matched wild-type siblings served as controls. All mice were maintained on the C57BL/6JRccHsd background. Food and water were provided *ad libitum*. The mice were terminally anesthetized with tribromoethanol (Avertin, Sigma-Aldrich, St. Louis, MO) followed by decapitation without transcranial perfusion. For each set of mice, cerebral cortex area was isolated, weighed and maintained on ice until isolation of lysosomal fractions. Animal experiments were approved by the national Animal Experiment Board of Finland and followed the animal protection guidelines of the Council of the European Union. Adult male and female wild-type (WT) zebrafish (AB strains) and the transgenic line Tg(neurod1:GCaMP6F) strain raised on the nacre (*mitfa*$^{-/-}$) background (a kind donation of Dr. Claire Wyart from Institut du Cerveau et de la Moelle Épinière, Paris, France) [55] were maintained according to standard procedures [56] on a 14 h light:10 h dark cycle. Whole mount *in situ* hybridization (WISH) was performed as described earlier [57] at the 24, 48, and 5dpf time points. For generation of zebrafish *cln5* knockdown, we designed morpholino antisense oligonucleotides (MOs) (GeneTools, Philomath, OR) targeting either the translation start site (i.e., atgMO) or transcription site at exon 1–2 splice site (i.e., spliceMO). Concentrations of MOs were carefully titrated to avoid nonspecific binding effects and a scrambled control MO was used at similar concentrations to assess specificity to *cln5*. Survival was calculated comparing death rates between microinjected and non-injected embryos.

RT-PCR

The effect of the MO was evaluated through RT-PCR. Primers for mRNA sequences were designed using the zebrafish sequence of *cln5* (ENSDART00000110866.4). Quantitative reverse transcription followed by the polymerase chain reaction in real time (qRT-PCR) expression analyses was done as described [29]. The MO and primers sequences are listed in **Supplementary Table S10**.

Sample preparation, and DIA-HDMS^E

Once demonstrated the high degree of enrichment provided by fractionation method (**Supplementary Figure S1**), ten µg of total protein from lysosomal fraction obtained from either HEK 293T cell lysate or mice cerebral cortex were digested using a modified FASP protocol as described [58]. Three-hundred nanograms of digested proteins (three technical replicates per sample) were used for DIA (Data Independent Acquisition) nano-liquid chromatography HDMS^E (High Definition Mass Spectrometry) analysis as described [59]. Database searches were carried out against human (release 2017_48461 entries) or *Mus musculus* (release 2017_16869 entries) UniProtKB/SwissProt, reviewed databases with ion accounting algorithm and using the following parameters: peptide and fragment tolerance: automatic, maximum protein mass: 500 kDa, minimum fragment ions matches per protein 7, fragment ions matches per peptide 3, peptide matches per protein 1, primary digest reagent: trypsin, missed cleavages allowed: 2, fixed modification: carbamidomethylation C, variable

modifications: NQ deamidation, oxidation of methionine (M) and false discovery rate (FDR) below 1%. Protein quantitation was performed entirely on non-conflicting protein identifications, using precursor ion intensity data and standardized expression profiles. The proteomics data were submitted to MassIVE (accession number MSV000088517)

Bioinformatic analysis and categorization of proteomic data

Differentially expressed proteins (DEP) were identified based on the number of unique peptides used for label-free quantitation (≥2), at the FDR<0.01 and the fold change (FC) from averaged, normalized protein intensities |≥1.3| for lysosomal mouse brains datasets and |≥1.5| for HEK 293T cell lines, utilizing $p \leq 0.05$ by ANOVA in all comparisons. Filtered datasets of differentially expressed proteins (DEPs) from both cellular and mouse models of CLN5 disease, were evaluated by QIAGEN's Ingenuity Pathway Analysis (IPA Winter Release - December 2020; QIAGEN, Hilden, Germany), to recognize meaningful biological processes and molecular pathways. Specifically, we carried out a *Core Analysis* followed by *Downstream Effects Analysis*, further scrutinizing the macro-categories of interest. P-value, which was ascertained by right-tailed Fisher's Exact Test following Benjamini and Hochberg (B-H) correction, indicated the robustness of correlation between a subset of DEGs of the dataset with a given biological function. Moreover, IPA algorithm calculated a ''z-score'' which estimates a predicted activation or inhibition of a given biological function. Only annotations with p-values <0.05 and activation z-scores >1.5 were taken into account in the bioinformatic analysis.

Enzyme activity assays

The activities of GLA (alpha-galactosidase, EC 3.2.1.22), GLB (beta-galactosidase-1, EC 3.2.1.23) and HEXB (the beta subunit of the enzyme hexosaminidase, EC 3.2.1.52) were determined using a fluorescence-based assay by employing a specific fluorogenic substrate for each enzyme involved in this study. Enzyme activity evaluation was based on hydrolysis of 4-methylumbelliferyl-α-D-galactopyranoside for GLA, 4-methylumbelliferyl-β-D-galactopyranoside for GLB, and 4-metylumbelliferyl N-acetyl-β-D-glucosaminide for HEXB, respectively [60–62]. All substrates were purchased from Santa Cruz Biotechnologies (Dallas, TX). To evaluate enzyme activity 20 µl for GLB and HEXB or 10 µl for GLA of each cellular/tissue lysate in duplicate were added to 20 µl of substrate and maintained at 37°C for 2h, 1h and 15min for HEXB, GLA and GLB respectively. Then 150 µl of Stop Buffer was added to each sample and fluorescence was measured with a Glomax Discovery microplate (Promega, Milano, IT) with an excitation filter 365 nm and emission filter 415–445 nm. Fluorescent signals were normalized by total protein amount using a BCA assay, comparing the absorbances at 562 nm with a BSA standard.

Western blotting

For western blotting, samples were homogenized in RIPA buffer (150 mM NaCl, 50 mM Tris-HCl, 6 mM EDTA, 1% NP-40, 0.1% SDS, 0.5% deoxycholic acid, pH 8.0) containing inhibitors of proteases (Roche Diagnostics GmbH, Mannheim, Germany) and centrifuged for 10 min at 14000×*g* at 4 °C. In all, 15–50 µg of protein lysates, determined by BCA assay (Invitrogen-ThermoFisher Scientific, Waltham, MA) was denatured and separated by electrophoresis using 8–16% Tris-Glycine Mini Gels (Invitrogen-ThermoFisher Scientific) and then electro-blotted onto PVDF membranes (Bio-Rad Laboratories Inc., Hercules, CA). Membranes were blocked with TBS/0.1%-Tween20 (TTBS) containing 5% non-fat dry milk before overnight incubation with the specified antibodies. Peroxidase-conjugated anti-mouse and anti-rabbit secondary antibodies (Jackson ImmunoResearch, Laboratories Inc., Cambridge, United Kingdom) were added for 1 h at room temperature in the same buffer as used for the primary antibodies (2.5% non-fat dry milk in TTBS). Reactive bands were detected using Clarity Max™ Western ECL Substrate (Bio-Rad Laboratories Inc., Hercules, CA), according to the manufacturer's instructions. Densitometry of Western blot bands was performed with the ImageJ software. Primary antibodies used for Western blotting analysis were as follows: rabbit monoclonal anti-CLN5 (Abcam #ab170899; dilution 1:1000), mouse monoclonal anti-LAMP2 (Abcam #ab25631; dilution 1:500), rabbit polyclonal anti-STX8 (Abcam #ab102798; dilution 1:500), rabbit monoclonal anti-GLA (Elabscience, Houston, TX; Cat #ESM20817; dilution 1:200), rabbit polyclonal anti-HEXB (Elabscience; Cat #EPP13904; dilution 1:200), rabbit monoclonal anti-TMEM106B (Abcam, Cambridge, United Kingdom; Cat #ab264323; dilution 1:500),

SCAMP3, (Invitrogen; Cat# PA5-21428, dilution 1:1000), LONP1, (Invitrogen; Cat# PA5-100020, dilution 1:1000). Immunodetection with rabbit polyclonal anti-β-tubulin antibody (Cell Signaling, danverse, MA; Cat #2146; dilution 1:2000) served as a loading control to normalize the bands intensity. For the zebrafish model, western blotting was performed on 2dpf zebrafish larvae as previously described [63], using the antibody against anti-microtubule-associated proteins 1A/B light chain 3B precursor (LC3B) (Sigma-Aldrich, Milan, Italy, Cat L7543, dilution 1:500) and as control anti-β-tubulin (Cell Signaling; Cat #2146, , dilution 1:1000).
For the drug treatment, embryos at 4hpf were selected and treated with miglustat and trehalose diluted in egg water to the final administered concentrations of 150 μM and 100 μM respectively [64]. A preliminary test was run on WT embryos to verify the non-toxic effect of substances. Mitochondrial respiration was analyzed in WT and morphant embryos at 96hpf using the XF24 extracellular flux analyzer (Seahorse Bioscience, North Billerica, MA.) as described in [65].

Immunofluorescence Microscopy
For immunofluorescence, the following antibodies were used: rabbit monoclonal anti-TMEM106B (Abcam; Cat #ab264323, dilution 1:100), rabbit polyclonal anti SCAMP3, (Invitrogen; Cat# PA5-21428, dilution 1:200), mouse monoclonal anti-CD107A (LAMP1) (BD Pharmigen, Bath, United Kingdom; Cat# 555798, dilution 1:1000). Goat anti-mouse and anti-rabbit conjugated with AlexaFluor 488 or AlexaFluor 555 dyes (Molecular Probes, Eugene, OR; dilution 1:1000) were used as secondary antibodies for 1 h at RT. Finally, nuclei were counterstained with 5 μg/ml DAPI (4′,6-diamidino-2-phenylindole dihydrochloride, Sigma-Aldrich). Cells were fixed in PFA 4% for 15 min and permeabilized in 0.1% (w/v) Triton X-100. Cells were incubated with indicated primary antibodies overnight, and subsequently probed with secondary antibodies including DAPI for 1 h. For confocal imaging, the samples were examined under a Zeiss LSM 900 confocal microscope. Optical sections were obtained under a × 63 immersion objective at a definition of 1,024 × 1,024 pixels (average of eight scans), adjusting the pinhole diameter to 1 Airy unit for each emission channel to have all the intensity values between 1 and 254 (linear range). For image analysis, a colocalization mask was generated directly by Zeiss ZEN digital imaging software suite (Zeiss, Oberkochen, germany), and areas were analyzed by ImageJ software [24]

Statistical analyses
Data presentation and statistical analyses were performed using Prism 8 (GraphPad Software, San Diego CA). For functional studies, data were presented stating *n* as a mean ± standard deviation (SD) from at least three independent experiments. Statistical analyses utilized Student *t* test with significance set at $P < 0.05$ (*), $P < 0.01$ (**), and $P < 0.001$ (***). For proteome analyses, six different lysosomal fractions from two distinct *CLN5* KO genotypes were compared with three mitochondrial samples obtained from a Ctrl cell line. Each biological replicate was analyzed in technical triplicates. To compare the controls and KO lines, we utilized the between-subject design scheme of the Progenesis QI™ software (Nonlinear Dynamics, Durham, NC). The ANOVA calculation applied by this scheme assumes that the conditions are independent and used a statistical test, which presumes that means of the conditions are equal and the variance similar between the groups. *P* values ≤ 0.05 by ANOVA were considered as significant. For studies in zebrafish, all data were analyzed by applying either parametric or non-parametric tests. Homogeneity of variance was assessed using the Levene test. Post hoc comparisons were performed by Mann-Whitney test, or Unpaired t-test following non-parametric analysis of variance. ANOVA with Tukey's Multiple Comparison Test and Fisher's exact and Chi-square test were used in specific zebrafish experiments.
