# Supplementary material for: Lysosomal Proteomics Links Disturbances in Lipid Homeostasis and Sphingolipid Metabolism to CLN5 Disease
Source: Cells. 2022 Jun 4;11(11):1840. doi: 10.3390/cells11111840 (PMC9180748; doi:10.3390/cells11111840)
Supplement: Supplementary file 1 [file cells-11-01840-s001.zip › Supplementary figures_final_rev_ML- VERY LAST.pdf]

Supplementary Figure S1

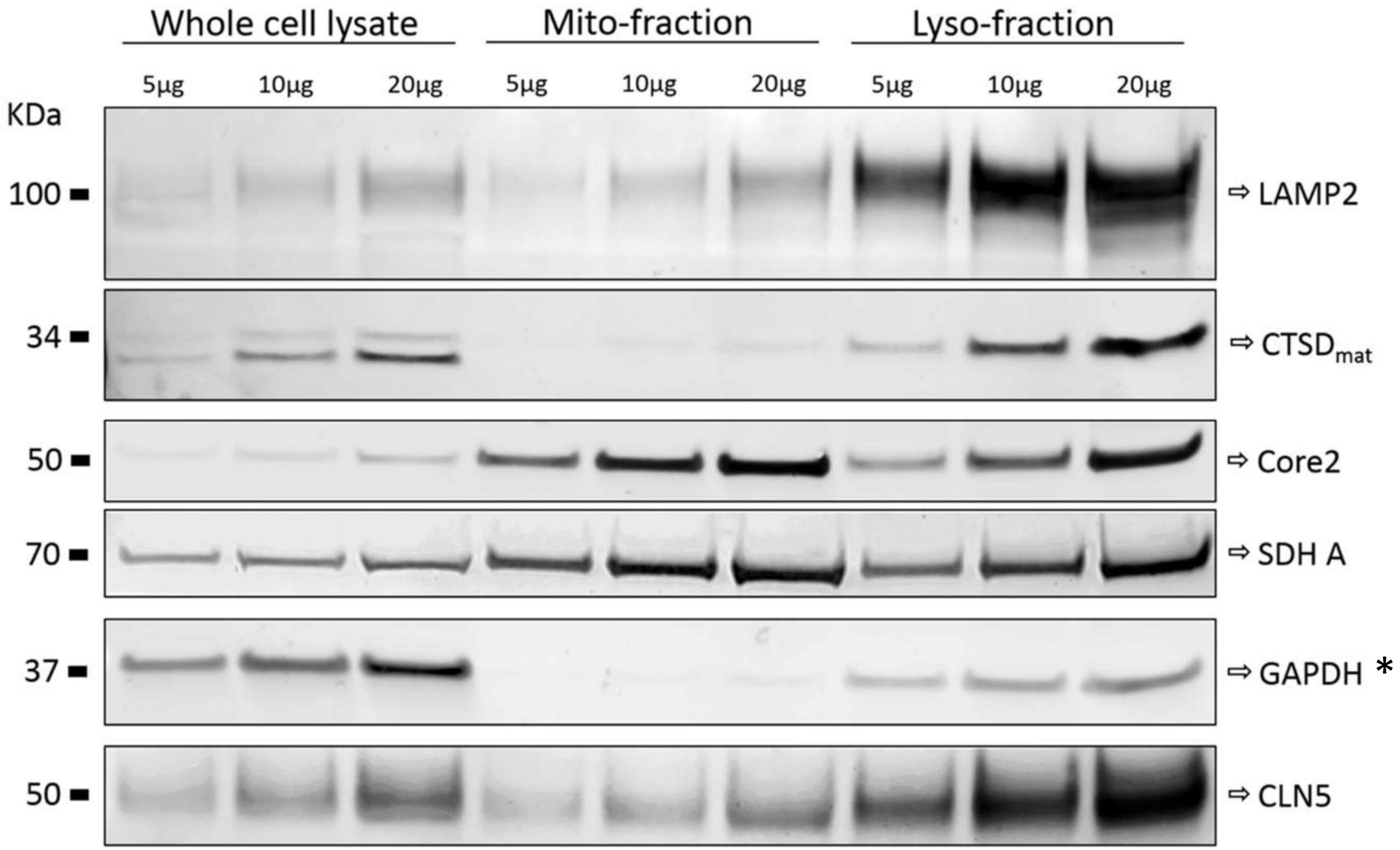

Supplementary figure S1. Monitoring the degree of enrichment and contamination level in mitochondrial and lysosomal preparations. Fractions from wildtype SH-SY5Y cells were probed with antibodies against lysosomal proteins (CTSD and LAMP2, representing soluble and membrane lysosomal markers respectively), mitochondria (SDHA and Core2). High degree of lysosomal enrichment can be observed as judged by LAMP2 and CTSDmat (mature form of CTSD) immunoreactivity, although the presence of mitochondrial markers in lysosome-enriched fractions indicates possible mitochondrial contamination. * Several studies demonstrated that the cytosolic enzyme GAPDH has pleiotropic functions independent of its canonical role in glycolysis. The subcellular and glycolytic-independent redistribution of the enzyme can mainly occur in the nucleus, mitochondria, and in early and late endosomes [Chauhan A.S. *et al*. *FASEB J.* 2019, doi:10.1096/fj.201802102R; Butera G. *et al*. *Int J Mol Sci.* 2019, doi: 10.3390/ijms20092062].

Supplementary Figure S2

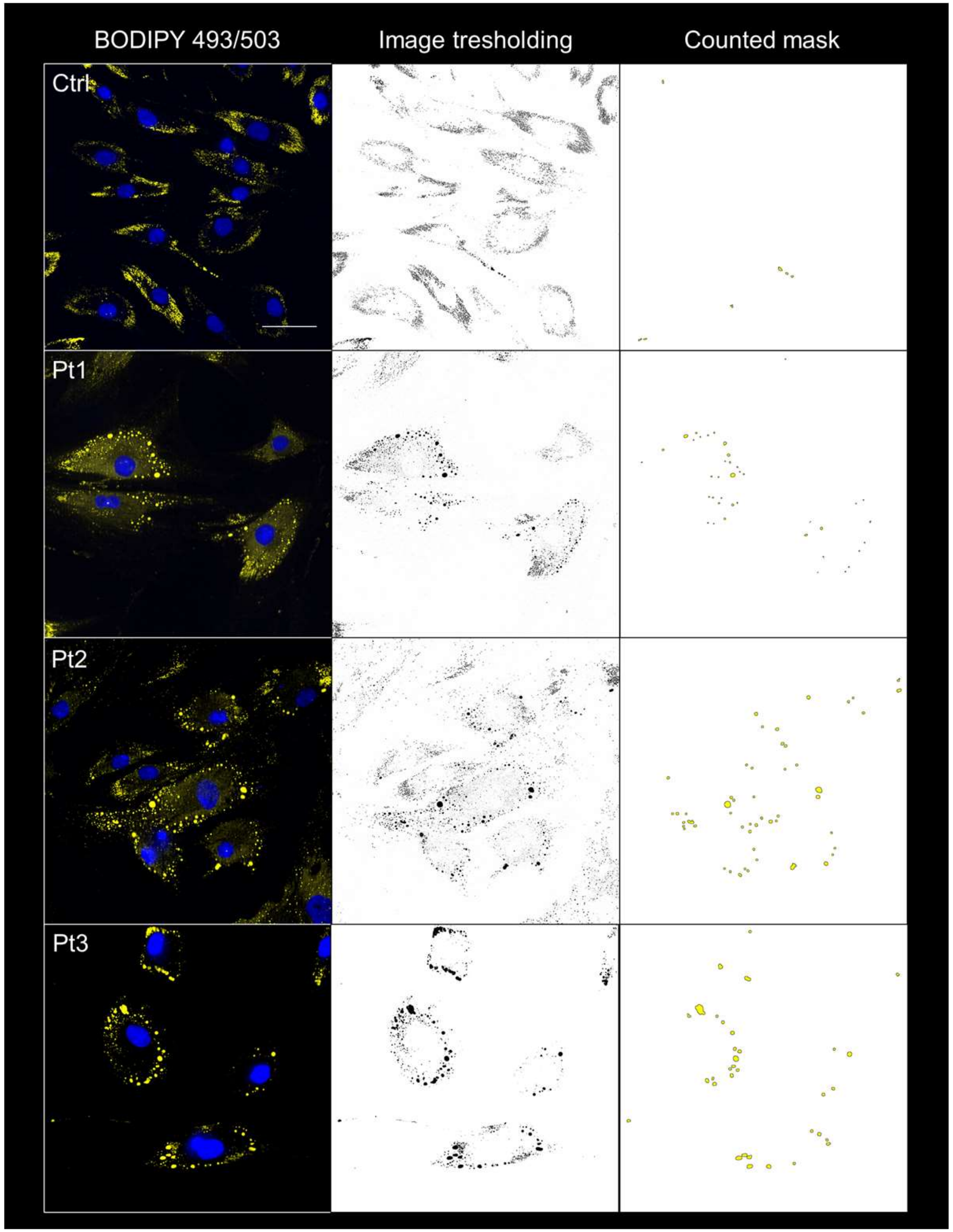

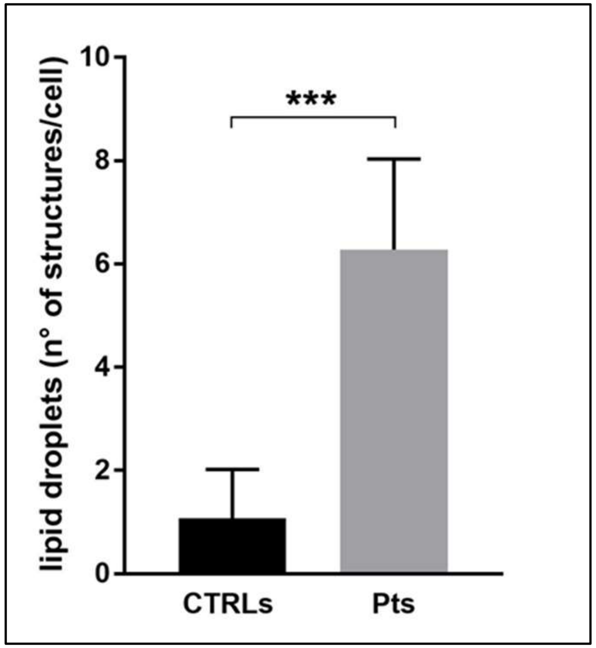

Supplementary Figure S2. Unbiased method for detection and quantification of intracellular lipids. Raw green channel (bodipy 493/503) was processed by applying a local thresholding and then analyzed by the “analyze particles” command of the ImageJ software. To select and count the structures resembling droplets-like in each field, we generated a count mask, setting the parameters of size and circularity. Objects with circularity > 0.6 with and area greater than 3 μm^2 were counted and normalized per cell number. At least 50 cells per condition was analyzed and data were reported by an histogram as mean ± SD. A significant increase (~ 6 fold) in the number of droplets-like structures was detected in patients’ cells (see graph).

Supplementary Figure S3

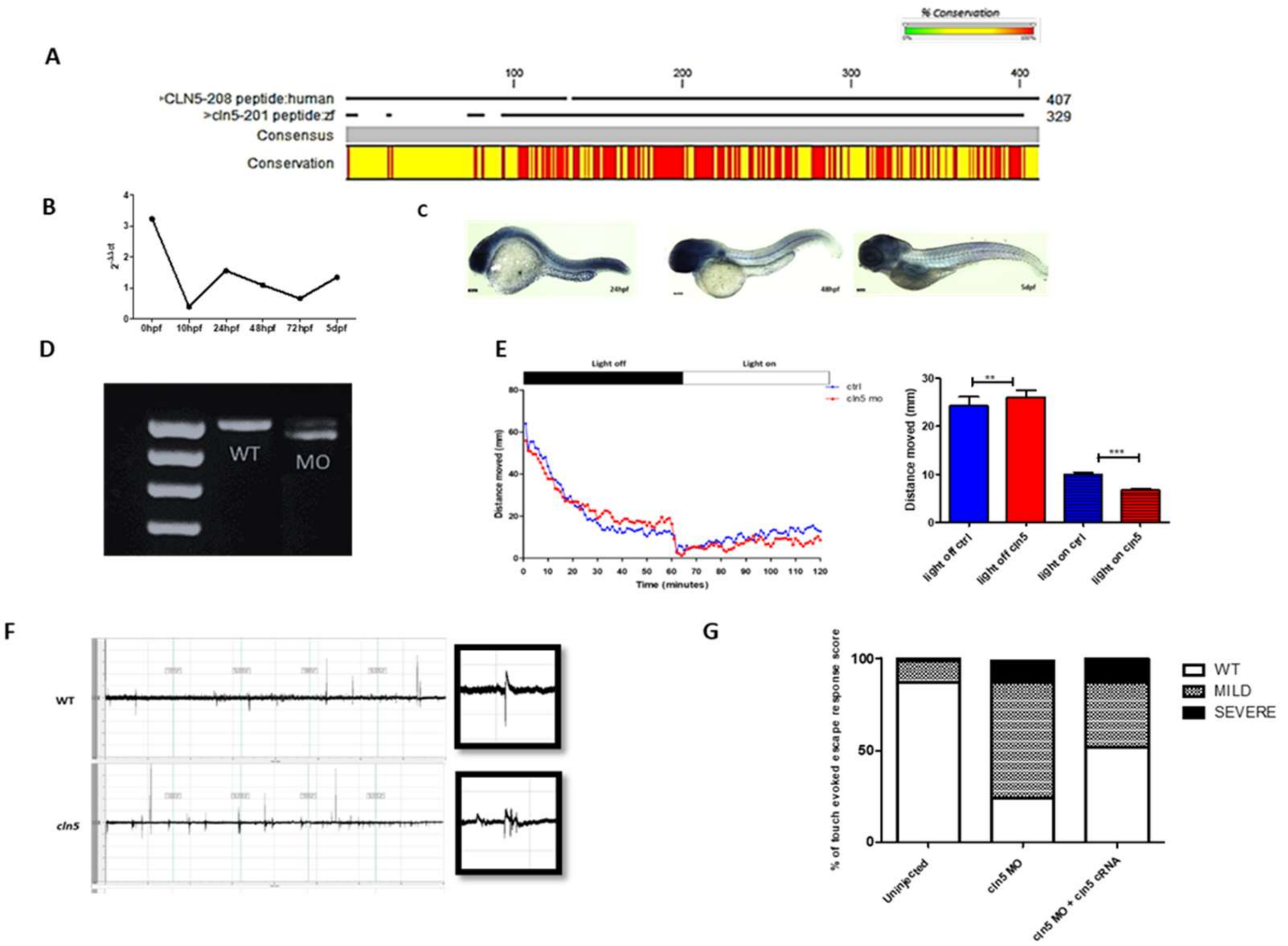

Supplementary Figure S3. Analysis of zebrafish CLN5 ortholog. (**A**) Bioinformatics analysis of a representative protein alignment of cln5 zebrafish form with its human ortholog. The percentage of conservation is around 70%. (**B**) Quantitative real-time PCR expression of zebrafish cln5 in different stages. (**C**) Whole mount in situ hybridization of *cln5*. (**D**) RT-PCR of *cln5* coding sequence on wild-type (WT) and morphants (MO) cDNAs. (**E**) Visual motor response test of *cln5* morphants (*cln5* MO) and control siblings (WT). (**F**) Local field potentials (LFP) recordings comparing morphants (*cln5*) and control siblings (WT), the inset in the black square represents the magnification of events, such as movement artifact in WT and seizures in *cln5* morphants (*cln5* MO). Methodological details are reported in Cozzolino O. *et al*. *Cells* 2020, doi: 10.3390/cells9030769. (**G**) Analysis of *cln5* splice MO rescue using WT *cln5* cRNA, through touch evoked response test. ***p ≤ .0001, **p < .001, according to Mann–Whitney test. Methodological details are reported in D'Amore A. *et al*. *Ann Clin Transl Neurol.* 2020, doi:10.1002/acn3.51018.

Supplementary Figure S4

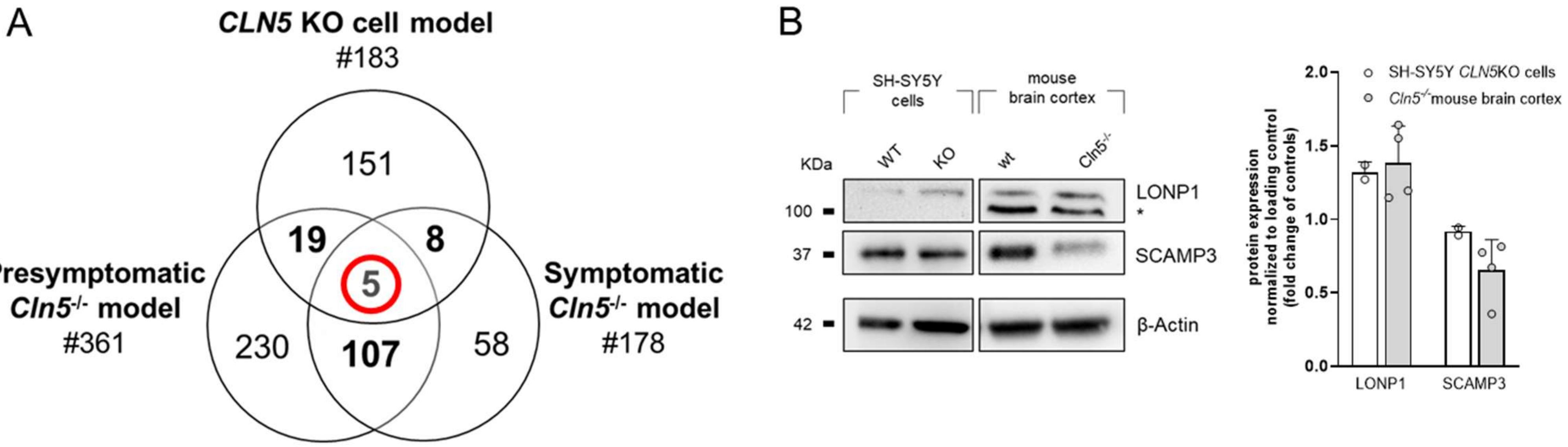

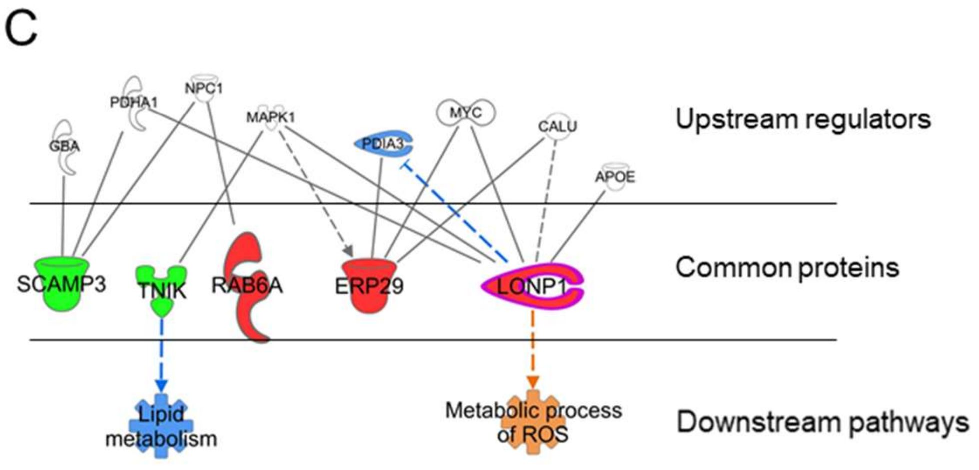

PROTEIN	cells	pre-sympt	sympt
TNIK	↓ -0.87	↑ 1.19	↓ -0.64
SCAMP3	↑ 0.36	↓ -0.83	↓ -2.74
RAB6A	↑ 0.57	↑ 1.28	↑ 1.56
LONP1	↑ 0.51	↑ 2.13	↑ 1.63
ERP29	↓ -0.72	↑ 0.87	↑ 1.34

Predictor legend

- Increased measurement
- Predicted activation
- ↑ Up-regulation (increased $\log_2$FC)
- Decreased measurement
- Predicted inhibition
- ↓ Down-regulation (decreased $\log_2$FC)

Supplementary figure S4. Lysosomal DEP identified across cellular and murine models (**A**) Venn diagram presenting lysosomal DEP identified in cellular and brain knockout models. Across all models, 5 dysregulated proteins involved in protein transport (RAB6A, ERP29, SCAMP3) and ATP binding (TNIK, LONP1) were identified. (**B**) LONP1 and SCAMP3 abundance levels were assayed by western blot analysis. (**C**) An involvement of these proteins in *Lipid metabolism* and *Metabolic process of ROS* was predicted by querying the IPA database (Qiagen, Hilden – Germany). Values reported in the table refer to $\log_2$ average fold change in protein abundance between KO and WT samples.
